# Common mental disorders among health workers: prevalence, co occurrence, and associated factors

**DOI:** 10.47626/1679-4435-2024-1260

**Published:** 2025-01-07

**Authors:** Natália Nascimento Silva, Saulo Vasconcelos Rocha, Rosângela Souza Lessa, Fernanda Queiroz Rego de Sousa Lopes, Clarice Alves dos Santos

**Affiliations:** 1 Department of Philosophy and Human Sciences, Universidade Estadual do Sudoeste da Bahia (UESB), Vitória da Conquista, BA, Brazil; 2 Department of Health II, UESB, Jequié, BA, Brazil; 3 Faculdades Santo Agostinho, Vitória da Conquista, BA, Brazil; 4 Universidade Estadual de Feira de Santana, Feira de Santana, BA, Brazil; 5 Department of Biological Sciences, UESB, Jequié, BA, Brazil

**Keywords:** primary health care, health personnel, mental health, atenção primária à saúde, trabalhadores da saúde, saúde mental

## Abstract

**Introduction:**

Common mental disorders are characterized as a set of symptoms that cause significant functional disability.

**Objectives:**

To assess the prevalence and clustering of the main symptoms of common mental disorders and the association between sociodemographic/occupational variables and common mental disorders among primary health care workers in the Brazilian cities of Vitória da Conquista, Bahia, and São Geraldo da Piedade, Minas Gerais.

**Methods:**

This cross-sectional survey is part of the Longitudinal Study of Physical Activity and Health of Workers in the Health Sector. The Self-Reporting Questionnaire was used to assess common mental disorders. Descriptive statistics, the chi-square test, cluster analysis, and binary logistic regression were performed in IBM SPSS Statistics 22.0.

**Results:**

A total of 107 primary health care workers from Vitória da Conquista (n = 92) and São Geraldo da Piedade (n = 15) participated. The prevalence of common mental disorders was 36.4%, with the most commonly reported symptoms being: feeling nervous, tense, or worried (51.4%); easily fatigued (46.7%); poor sleep (43.9%); frequent headaches (36.4%); and recent feelings of sadness (36.4%). Approximately one-third of the workers had common mental disorders, with 5 simultaneous symptoms being the most prevalent combination. Workers with lower education and those who lived with a partner had a higher and lower risk of common mental disorders, respectively.

**Conclusions:**

Identifying the prevalence and the main symptoms associated with common mental disorders can facilitate the development and implementation of more targeted and effective care actions for this population.

## Abstract (pt)

**Introdução:**

Os transtornos mentais comuns caracterizam-se como um conjunto de sintomas que provocam incapacidade funcional significativa.

**Objetivos:**

Avaliar a prevalência e o agrupamento dos principais sintomas de transtornos mentais comuns, bem como a associação entre aspectos sociodemográficos, ocupacionais e a presença de transtornos mentais comuns entre trabalhadores da Atenção Primária à Saúde.

**Métodos:**

Trata-se de um levantamento de delineamento transversal oriundo do Estudo Longitudinal de Atividade Física e Saúde dos Trabalhadores do Setor Saúde. Foi utilizado o Self-Reporting Questionnaire para avaliar os transtornos mentais comuns. Na análise dos dados, foram utilizados procedimentos da estatística descritiva, teste qui-quadrado e análise de *cluster* e regressão logística binária, realizada no *software* Statistical Package for the Social Sciences 22.0.

**Resultados:**

No total, participaram 107 trabalhadores da Atenção Primária à Saúde dos municípios de Vitória da Conquista, estado da Bahia (n = 92), e São Geraldo da Piedade, estado de Minas Gerais (n = 15). A prevalência de transtornos mentais comuns foi de 36,4%, e os sintomas mais relatados pelos trabalhadores foram: sente-se nervoso(a), tenso(a) ou preocupado(a) (51,4%); cansa-se com facilidade (46,7%); dorme mal (43,9%); tem dores de cabeça frequentemente (36,4%); e tem se sentido triste ultimamente (36,4%). Aproximadamente um terço dos trabalhadores apresentou transtornos mentais comuns, sendo a combinação mais prevalente a presença de cinco sintomas simultaneamente. Trabalhadores com menores níveis de escolaridade e aqueles que viviam com companheiros tinham maior e menor risco de apresentar transtornos mentais comuns, respectivamente.

**Conclusões:**

A identificação da prevalência e dos principais sintomas associados aos transtornos mentais comuns pode colaborar na elaboração e implementação de ações de cuidado mais direcionadas e efetivas para esta população.

## INTRODUCTION

Work can promote health, personal satisfaction and socialization through interpersonal and transpersonal relationships.^1^ However, when workers find an unfavorable environment and see no prospects for growth or professional advancement, work can become a source of illness.^2^ Susceptibility to illness may also increase due to risk factors such as high emotional demands, precarious contracts, and conflict between personal and professional life.^2^

Common mental disorders (CMD) are characterized as a set of symptoms, such as insomnia, fatigue, forgetfulness, difficulty concentrating, and somatic complaints, that cause significant functional incapacity. They cause psychosocial damage to the patient and incur high social and economic costs.^3^

Prevalence findings for CMD among primary health care (PHC) workers in Brazil range from 16% in the Northeastern and Southern regions^4^ to 42.6% in the state of São Paulo.^5^ Occupational characteristics, such as job dissatisfaction,^6^ work hours, work complexity, and management relations^7^ can increase the vulnerability to these morbidities. Sociodemographic characteristics such as sex,^6,7^ race,^8^ age,^3,9^ education,^3^ and income^3^ can also contribute to the increased occurrence of CMD.

The Self-Reporting Questionnaire (SRQ 20) is among the most important CMD tracking instruments.^10^ The SRQ-20 has been validated and translated for use in Brazil^11^ and consists of 20 dichotomous questions on 4 groups of symptoms: depressive/anxious mood, somatic symptoms, decreased vital energy, and depressive thoughts.^12^ Although investigations into CMD prevalence are already common in epidemiological surveys, studies assessing these dimensions are scarce^10^ and surveys that analyze the simultaneity or clustering of symptoms are even rarer. Understanding the influence of these dimensions and how symptoms cluster can help identify the greatest risk factors for CMD among these workers, which can lead to more effective care actions and/or interventions.

Thus, the objective of the present study was to evaluate the clustering of the main symptoms of CMD, as well as the association between sociodemographic/occupational characteristics and CMD prevalence among PHC workers.

## METHODS

This cross-sectional epidemiological study is based on data from the Longitudinal Study of the Physical Activity and Health of Health Sector Workers (*Estudo Longitudinal de Atividade Física e Saúde dos Trabalhadores do Setor Saúde*), which was approved by the Universidade Estadual do Sudoeste da Bahia ethics committee (decision 3,560,194) according to Brazilian National Health Council resolution 466/2012. The study was conducted in the municipalities of Vitória da Conquista, Bahia, and São Geraldo da Piedade, Minas Gerais among health professionals associated with the local primary care network.

Vitória da Conquista, which is in southwestern Bahia, has an area of 3,254.186 km^2^ and an estimated population of 343,643.^13^ In its metropolitan area, there are 16 basic health units and 38 Family Health Program teams (which cover 63% of the population).^14^ São Geraldo da Piedade, which is 209.09 km northeast of the state capital Belo Horizonte, has an area of 152.336 km^2^ and an estimated population of 3,860.^13^ The municipality has two basic health units and 5 family health strategy teams (which cover 100% of the population).^15,16^

A census was conducted of primary care workers in these municipal health systems. Employee lists provided by the Municipal Health Departments were checked at the workplaces, and all PHC workers were invited to participate. Due to the ongoing COVID-19 pandemic, data were collected online through a 2-stage electronic questionnaire regarding the following variables: sociodemographic data (sex, age in years, monthly income, race, education level, and marital status), occupational data (weekly work hours, employment type, and multiple employment) and CMD. CMD prevalence was assessed using the SRQ-20, a 20-item self-report questionnaire on somatic and psychological factors. This instrument is recommended by the World Health Organization to identify mental health problems in populations in developing countries.^17^ Based on a Brazilian validation study, SRQ-20 scores ≥ 7 points were considered indicative of potential CMD.^11^ For the analysis, CMD symptoms were grouped according to the SRQ-20 dimensions (depressed/anxious mood, somatic symptoms, decreased vital energy, and depressive thoughts). Descriptive statistics, association measures for categorical variables (chi-square test), cluster analysis, and binary logistic regression were performed in IBM SPSS Statistics 22.0.

## RESULTS

A total of 107 PHC workers from Vitória da Conquista (n = 92) and São Geraldo da Piedade (n = 15) participated in the study. The mean participant age was 38.91 (SD, 11.679) years. Most were women (83.2%), lived with a partner (88.8%), were Black or of mixed race (83.2%), and had higher education (61.7%). Regarding occupational characteristics, most were public servants (79.4%), worked ≥ 40 hours each week, and did not have another job (81.3%). The sociodemographic and occupational characteristics and their association with CMD are shown in Table 1.

**Table 1:** Sociodemographic and occupational characteristics of health professionals with and without common mental disorders (CMD)

Variables	Total sample n (%)	Without CMD n (%)	With CMD n (%)	p-value
Sex				
Female	89 (83.2)	54 (50.5)	35 (32.7)	0.169
Male	18 (16.8)	14 (13.1)	4 (3.7)	
Age range (years)				
≤ 41	52 (48.6)	33 (30.8)	19 (17.8)	0.985
≥ 42	55 (51.4)	35 (32.7)	20 (18.7)	
Marital status				
Without partner	12 (11.2)	3 (2.8)	9 (8.4)	0.003
With partner	95 (88.8)	65 (60.7)	30 (28.0)	
Education				
Higher education	66 (61.7)	48 (44.9)	18 (16.8)	0.012
≤ High school	41 (38.3)	20 (18.7)	21 (19.6)	
Income				
≤ Minimum wage	21 (19.6)	13 (12.1)	8 (7.5)	0.861
> Minimum wage	86 (80.4)	55 (51.4)	31 (29.0)	
Race				
White/Asian	18 (16.8)	12 (11.2)	6 (5.6)	0.763
Black/mixed	89 (83.2)	56 (52.3)	33 (30.8)	
Weekly work hours				
≤ 39	20 (18.7)	12 (11.2)	8 (7.5)	0.714
≥ 40	87 (81.3)	56 (52.3)	31 (29.0)	
Employment relationship				0.613
Public servant	85 (79.4)	53 (49.5)	32 (29.9)	
Officially employed, outsourced, or positions of trust	22 (20.6)	15 (14.0)	7 (6.5)	
Multiple employment				0.714
No	87 (81.3)	56 (52.3)	31 (29.0)	
Yes	20 (18.7)	12 (11.2)	8 (7.5)	

The overall prevalence of CMD was 36.4%, with the most reported symptoms being: feeling nervous, tense, or worried (51.4%); easily fatigued (46.7%); poor sleep (43.9%); frequent headaches (36.4%); and recent feelings of sadness (36.4%), as shown in Table 2.

**Table 2:** Characteristics of the dimensions of common mental disorders among health care workers

Symptom groups	Yes n (%)	No n (%)
Depressive/anxious mood		
Feeling nervous, tense, or worried	55 (51.4)	52 (48.6)
Easily scared	37 (34.6)	70 (65.4)
Recent feelings of sadness	39 (36.4)	68 (63.6)
Crying more than usual	16 (15.0)	91 (85.0)
Somatic symptoms		
Frequent headaches	39 (36.4)	68 (63.6)
Poor sleep	47 (43.9)	60 (56.1)
Unpleasant stomach sensations	30 (28.0)	77 (72.0)
Poor digestion	30 (28.0)	77 (72.0)
Appetite loss	20 (18.7)	87 (81.3)
Hand tremors	17 (15.9)	90 (84.1)
Decreased vital energy		
Easily fatigued	50 (46.7)	57 (53.3)
Decision-making difficulties	36 (33.6)	71 (66.4)
Difficulty performing daily activities satisfactorily	30 (28.0)	77 (72.0)
Work becoming increasingly difficult	18 (16.8)	89 (83.2)
Feeling tired all the time	33 (30.8)	74 (69.2)
Difficulty thinking clearly	27 (25.2)	80 (74.8)
Depressive thoughts		
Inability to play a useful role in one’s own life	10 (9.3)	97 (90.7)
Losing interest in things	25 (23.4)	82 (76.6)
Suicidal thinking	5 (4.7)	102 (95.3)
Feeling useless and worthless	12 (11.2)	95 (88.8)

Table 3 presents the observed (O) prevalence, expected (E) prevalence and the O/E ratio for the 32 combinations of the 5 most common symptoms. The most prevalent combinations were the presence of all 5 symptoms (feeling nervous, tense, and worried; frequent headaches; recent feelings of sadness; poor sleep; and easily fatigued) (O = 19.6; E = 1.4; O/E = 14.04), followed by the absence of all 5 (O = 28; E = 5.9; O/E = 4.76).

**Table 3:** Prevalence of combinations of symptoms of the 5 most common mental disorder reported by health care workers

Factors	NER	FHA	SAD	PSL	EFA	O (%)	E (%)	O/E
5	+	+	+	+	+	19.6	1.4	14.04
4	-	+	+	+	+	3.7	1.3	2.80
4	+	-	+	+	+	0.0	2.4	0.00
4	+	+	-	+	+	0.0	2.4	0.00
4	+	+	+	-	+	4.7	1.8	2.63
4	+	+	+	+	-	3.7	1.6	2.32
3	-	-	+	+	+	0.0	2.3	0.00
3	-	+	-	+	+	0.0	2.3	0.00
3	-	+	+	-	+	0.0	1.7	0.00
3	-	+	+	+	-	0.0	1.5	0.00
3	+	-	-	+	+	3.7	4.3	0.87
3	+	-	+	-	+	0.0	3.1	0.00
3	+	-	+	+	-	0.0	2.8	0.00
3	+	+	-	-	+	0.0	3.1	0.00
3	+	+	-	+	-	0.0	2.8	0.00
3	+	+	+	-	-	2.8	2.0	1.37
2	-	-	-	+	+	6.5	4.0	1.61
2	-	-	+	-	+	0.0	2.9	0.00
2	-	-	+	+	-	0.0	2.6	0.00
2	+	-	-	-	+	1.9	5.4	0.35
2	+	-	-	+	-	4.7	4.9	0.97
2	+	-	+	-	-	0.0	3.6	0.00
2	+	+	-	-	-	0.0	3.6	0.00
2	-	+	-	-	+	0.0	2.9	0.00
2	-	+	-	+	-	0.0	2.6	0.00
2	-	+	+	-	-	0.0	1.9	0.00
1	-	-	-	-	+	6.5	5.2	1.26
1	-	-	-	+	-	1.9	4.6	0.41
1	-	-	+	-	-	0.0	3.4	0.00
1	+	-	-	-	-	10.3	6.2	1.66
1	-	+	-	-	-	0.0	3.4	0.00
0	-	-	-	-	-	28.0	5.9	4.76

E = expected prevalence; FHA = frequent headaches; NER = feeling nervous, tense, or worried; O = observed prevalence; O/E = ratio between observed and expected prevalence; PSL = poor sleep; SAD = recent feelings of sadness; EFA = easily fatigued.

As shown in Table 4, variables with p < 0.20 in the crude analysis (sex, marital status, and education) and those associated with CMD based on the assumptions in the literature (weekly work hours and multiple employment) were included in the model. According to the results, those who lived with a partner and those with lower education were less and more likely to have CMD, respectively.

**Table 4:** Association of independent variables with the presence of common mental disorders (regardless of symptoms) among health care workers

Variables	OR_crude_ (95%CI)	OR_adjusted_ (95%CI)
Sex		
Female	1	1
Male	0.44 (0.13-1.44)	0.36 (0.10-1.31)
Marital status		
With partner	1	1
Without partner	0.15 (0.03-0.60)	0.18 (0.44-0.80)
Education		
Higher education	1	1
≤ High school	2.80 (1.23-6.34)	2.75 (1.10-6.83)
Multiple employment		
No	1	1
Yes	1.20 (0.44-3.26)	1.67 (0.52-5.28)
Weekly work hours		
≤ 39	1	1
≥ 40	0.83 (0.30-2.24)	0.78 (0.26-2.35)

OR = odds ratio.

## DISCUSSION

The present study assessed the prevalence of CMD, symptom clustering, and the association between CMD and sociodemographic and occupational variables, finding that approximately one-third of the participants had CMD. The most prevalent symptoms were feeling nervous, tense, or worried and being easily fatigued. Of the 32 combinations of CMD symptoms, 7 clusters were observed (O/E > 1.0). A combination of 5 symptoms had the highest cluster score (O/E = 14.04). According to multivariate analysis, workers who lived with a partner and those with a lower education level were less and more likely, respectively, to have a CMD. Although the CMD prevalence was high, it was similar to other epidemiological surveys of health professionals conducted in Bahia^18^ and in the city of Porto Alegre, Rio Grande do Sul.^12^ A number of factors could have contributed to these results, but the fact that the study was conducted during the COVID-19 pandemic may have influenced the prevalence of mental illness in this population through psychological repercussions, such as: fear, anxiety, stress, sleep disorders, and nervousness.^19^

At the most critical point of the pandemic (2020 and part of 2021), attention turned to the hospital context, which received greater concern and investment, as did the professionals who worked there.^20^ Although this attention has been important, little has been observed about the repercussions of this scenario on professionals in other levels of health care; few studies could be found on the repercussions of the COVID-19 pandemic among PHC workers.

From this perspective, occupational and environmental characteristics, combined with the pandemic, may have influenced the emergence/worsening of CMD symptoms.^19^ It is worth noting that basic health unit professionals work in close proximity to the community.^21^ Community health agents, for example, serve as a link between the community and the health unit, with home visits used as a means to this end.^22^ During the pandemic, such visits were considered risky, which further highlights the extreme importance of health professionals for affected populations.^22^ Therefore, the care these workers receive, especially regarding their mental health, must be considered.

In addition, basic health unit workers have direct contact with the public and must deal with technical demands and social issues, such as poverty and violence.^12^ Like community health agents, they too may often feel responsible for these difficulties the population faces.^23^ These and similar details make PHC workers susceptible to suffering and/or mental illness.

The most prevalent CMD symptoms in the present study have been found in other investigations of health professionals. According to Carlotto,^12^ 61% of primary care workers in Porto Alegre reported feeling nervous, tense, or worried, and similar findings were reported by Alves et al.^24^ in a survey of university hospital workers in Uberaba, Minas Gerais. However, the prevalence of easy fatigue in our sample was more pronounced than in those of Carlotto (2.6%)^12^ or Alves et al. (34.6%).^24^

The clustering of CMD symptoms has not been widely explored in the literature, and we found a higher prevalence of a combination of 5 symptoms, whether present or absent. Thus, it can be concluded that the presence of 1 symptom is associated with an increased risk of the others. In addition to a high prevalence of CMD, these workers are affected by physical, psychological, and emotional symptoms, which demonstrates their vulnerability to suffering and mental illness.

CMD were more prevalent among workers with lower education levels, and other surveys have found similar results.^18,25^ Evidence from the literature indicates that a higher education level increases access to knowledge of various kinds, helps develop cognitive skills, and contributes to more assertive behavior, decision-making, and independence.^25^ From this perspective, the concept of health literacy becomes useful, i.e., the ability to acquire, retain, and interpret basic health information that helps in appropriate decision-making.^26^

A higher education level is also associated with higher income, as years of study can qualify an individual for more important positions.^27^ Considering that the job market has become increasingly demanding regarding academic training, it is expected that unemployment levels will increase among those with less education. Higher income enables easier access to specialized health services, preventive medicine and medications, and increases the possibility of private health insurance.^28^ Income also influences health through access to quality housing and basic sanitation. Poor living conditions can increase the likelihood of disease.^28^ The Unified Health System can help eliminate these social inequalities by offering health education, preventive programs, and treatment to all Brazilians. Nevertheless, poor service conditions often affect user access, especially among the poorest, who seem to need the system the most.^28^

Our results also showed that workers who lived with a partner were less likely to suffer from a CMD. An unsatisfactory romantic relationship can cause several types of harm, such as an increased propensity to mental disorders and a heightened risk of violent acts and suicide.^29^ However, some studies have shown that having a partner is a protective factor for mental health, provided the relationship is healthy.^29^ It is important to highlight that the lack of a partner during the most critical period of the COVID-19 pandemic may have increased feelings of loneliness, thus contributing to CMD prevalence. Guilland et al.^30^ found higher anxiety levels among workers from various sectors who reported being single during the pandemic, pointing out that having someone to talk to can be a protective factor against stress and anxiety.^30^

No associations were observed between CMD and sex, which contrasts with the literature, since adult women^3^ and female health care workers have been reported to be more vulnerable to CMDs.^6,8^ Thus, although we did not find a significant association between sex and CMD, it is important to consider that factors such as double work shifts (i.e., professional and domestic work) can generally increase women’s vulnerability to health problems.^8^ The overload from combining work and domestic activities can increase women’s propensity for CMDs.^8^ Furthermore, Campos et al.^8^ found that domestic work falls disproportionately on women. The same survey identified that women participate less in leisure activities and physical activities than men. Hence, it can be concluded that women, due to more frequent double work shifts, may have less leisure time.

An important limitation is that this study’s cross-sectional design does not allow assessment of cause-and-effect relationships between the variables and the outcome. Self-reported data are also subject to recall bias. However, we were able to survey primary care workers from municipalities in different regions of the country during the COVID-19 pandemic, and our findings are the baseline results of a cohort study that aims to monitor these workers for 10 years.

## CONCLUSIONS

This study demonstrated that health care workers are vulnerable to CMD, as well as the most common CMD symptoms: feeling nervous, tense, or worried; being easily fatigued; having poor sleep; having frequent headaches; and recent feelings of sadness. Importantly, the simultaneity analysis showed that the presence of one symptom increases the probability of others. Finally, our results are generally in line with investigations involving more robust designs (cohort studies) and provide important information for new health care policies for health care workers.
